# Supplementary material for: The association between depressive symptoms and self-reported sleep difficulties among college students: Truth or reporting bias?
Source: PLoS One. 2021 Feb 19;16(2):e0246370. doi: 10.1371/journal.pone.0246370 (PMC7894923; doi:10.1371/journal.pone.0246370)
Supplement: S1 Appendix — (PDF) [file pone.0246370.s002.pdf]

S1 Appendix. Vignettes used in this study

Vignette 1

[Firstname][1] falls asleep every night within 5 minutes after having gone to bed. S/He sleeps deeply during the entire night and wakes up in the morning well-rested.

Vignette 2

[Firstname] does not have any problem falling asleep in the evening and does not wake up during the night but s/he has some difficulties waking up in the morning. S/He uses an alarm clock but falls back asleep after the alarm goes off. S/He is late to school 4 days out of 5.

Vignette 3

[Firstname] easily falls asleep in the evening, but two times per week, s/he wakes up in the middle of the night and can't fall back asleep again.

Vignette 4

[Firstname] wakes up about once every hour during the night. When s/he wakes up during the night, it takes her/him about 15 minutes to fall back asleep again. In the morning, s/he does not feel well-rested.

Vignette 5

[Firstname] takes about 2 hours to fall asleep every night. S/He wakes up once or twice per night, feeling panicked and it takes her/him more than an hour to fall back to sleep again.

After reading each vignette, the respondents were asked to answer the following question: *Overall in the last 30 days, how much of a problem did [Firstname] have with sleeping, such as falling asleep, waking up frequently during the night or waking up too early in the morning?*

[1]Note that the first names used in the vignettes were sex-specific.
