# Supplementary material for: The association between depressive symptoms and self-reported sleep difficulties among college students: Truth or reporting bias?
Source: PLoS One. 2021 Feb 19;16(2):e0246370. doi: 10.1371/journal.pone.0246370 (PMC7894923; doi:10.1371/journal.pone.0246370)

S3 Appendix. Response consistency

Response consistency assumes that respondents use the same scale to evaluate their own condition and the condition of the persons described in the vignettes. One implication of that assumption is therefore that respondents whose characteristics are similar to the characteristics of the hypothetical person described in the vignettes should have self-assessments that converge towards the assessment of the vignettes. We use this implication as an informal test to assess whether the response consistency holds in our setting. In particular, we first compare the distribution of self-assessed sleep difficulty with that of a vignette using our entire study sample. We then make the same comparison but restrict our sample to respondents whose characteristics are similar to the characteristics of the person described in the vignettes. If response consistency holds, the distribution of the self-assessments and that of the vignette evaluations should get closer to each other when imposing the sample restriction.

The matching between respondent characteristics and the characteristics of the person described in the vignettes is far from perfect as our online survey unfortunately does not contain all the particular sleep dimensions that are described in the vignettes. In fact, we can convincingly proceed to these comparisons only for vignettes 1 and 5, as we do not have information about whether our respondents wake up during the night (vignettes 2, 3 and 4), have difficulties waking up in the morning (vignette 2) or have difficulties falling back asleep when they wake up during the night (vignettes 3 and 4).

Although imperfect, we nonetheless proceed to these comparisons for vignettes 1 and 5. More specifically, vignette 1 describes the scenario of a person who is able to fall asleep within 5 minutes after having gone to bed. We therefore restrict the sample to respondents who fall sleep within the same time period. Similarly, vignette 5 describes the scenario of someone who takes more than two hours to fall asleep. We hence restrict our comparison to those who take the same amount of time to fall asleep. Again, these matchings are incomplete as we do not have any information about whether respondents feel well rested when they wake up in the morning (vignette 1) or whether they frequently wake up during the night and have issues falling back asleep again (vignette 5). But the comparisons we perform could represent suggestive evidence that response consistency holds in our setting.

Figures S3a and S3b present the results of these comparisons for vignettes 1 and 5, respectively. These plots show the cumulative distribution functions (CDFs) of self-assessed sleep quality and vignette evaluations for our entire sample and for the sub-sample of college students (labeled “restricted” in the legend of the figure) who match the characteristics of the hypothetical persons described in the vignettes. Even though the matchings are imperfect, our data support the convergence of the CDFs once we impose the sample restrictions. When respondents become more “similar” to the persons described in vignettes 1 and 5, their self-assessments converge to the vignette assessments, providing suggestive evidence that the response consistency assumption holds in our setting.

[INSERT FIGURE S3a HERE] **Response consistency - vignette 1**

Note: Vignette 1 describes the scenario of someone who is able to fall asleep within 5 minutes after having gone to bed. We therefore restrict the sample to respondents who fall sleep within the same time period. The evaluations of the individuals who are included in that restricted sample are labeled "restricted" in the legend of the figure. Solid line represents the self-assessed sleep difficulties for the entire sample.

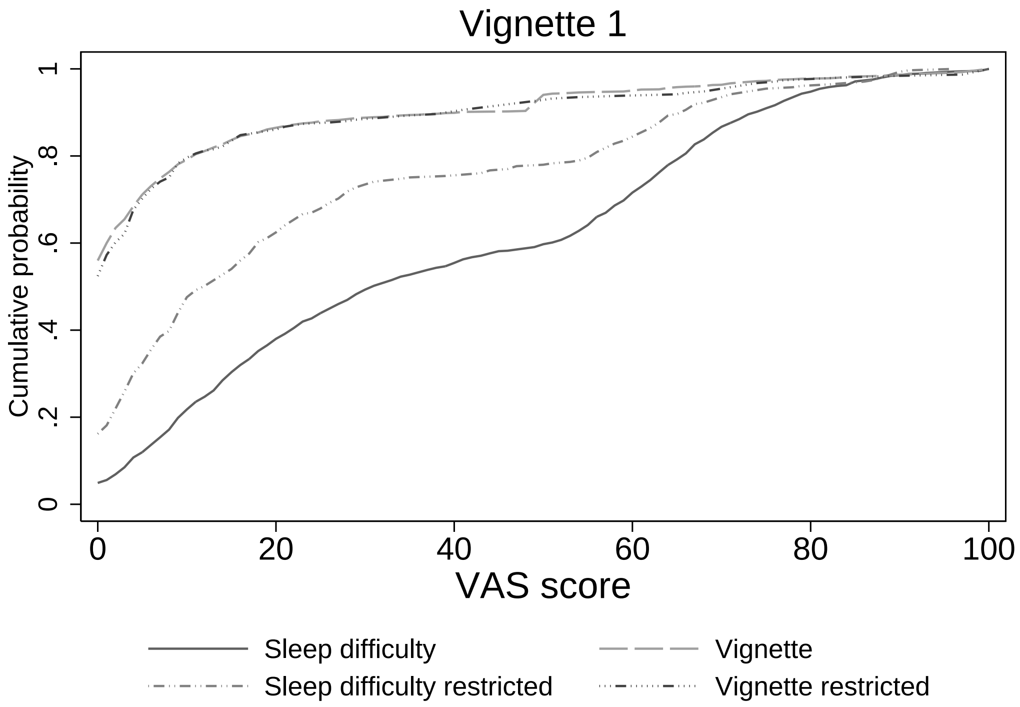

[INSERT FIGURE S3b HERE] **Response consistency - vignette 5**

Note: vignette 5 describes the scenario of someone who takes more than two hours to fall asleep. We therefore restrict our comparison to those who take the same amount of time to fall asleep. The evaluations of the individuals who are included in that restricted sample are labeled "restricted" in the legend of the figure. Solid lines represent the self-assessed sleep difficulties for the entire sample.

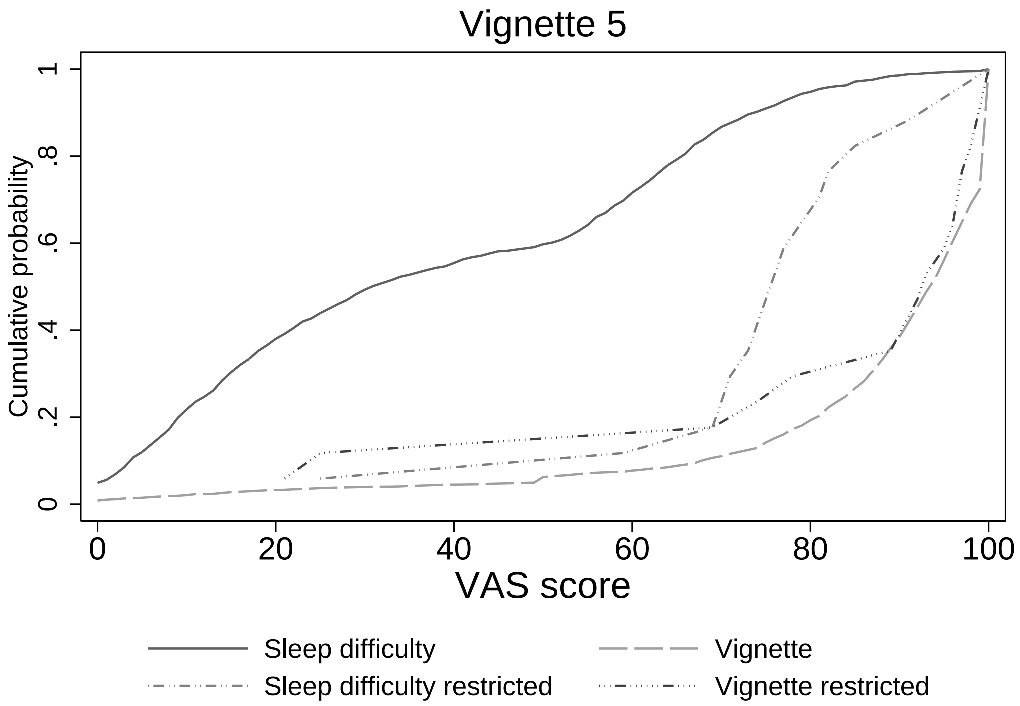

Supplement: S3 Appendix — (PDF) [file pone.0246370.s004.pdf]
