# Supplementary material for: The association between depressive symptoms and self-reported sleep difficulties among college students: Truth or reporting bias?
Source: PLoS One. 2021 Feb 19;16(2):e0246370. doi: 10.1371/journal.pone.0246370 (PMC7894923; doi:10.1371/journal.pone.0246370)
Supplement: S4 Appendix — (PDF) [file pone.0246370.s005.pdf]

S4 Appendix. Vignette equivalence

The vignette equivalence assumption states that vignettes are perceived in the same way by all respondents, up to an idiosyncratic error term. This means that the characteristics of the respondents do not affect the way respondents interpret the information that is given to them in the vignettes. One implication of vignette equivalence is that respondents would rank vignettes in the same order up to a measurement error. Most of the vignettes used in our study can be ranked "objectively" by level of sleep difficulties. Indeed, one can reasonably assume that the following ranking should prevail: $Vignette\,1 \leq Vignette\,\Gamma \leq Vignette\,4 \leq Vignette\,5$ with $\Gamma = \{2, 3\}$. Note that the ranking between vignettes 2 and 3 is not clear and we therefore do not consider $Vignette\,2 \geq Vignette\,3$ as an inconsistent ranking.

To test the implication of the vignette equivalence assumption, we first count the proportion of respondents who follow the ranking $Vignette\,1 \leq Vignette\,\Gamma \leq Vignette\,4 \leq Vignette\,5$ with $\Gamma = \{2, 3\}$. We then calculate the size of the vignette ranking inconsistencies by computing the difference (distance) in the VAS score for the vignettes that were incorrectly ranked. Finally, we re-estimate all our models after dropping respondents who have "incorrectly" ranked the vignettes as robustness check.

The table below shows that up to 68.9% of respondents have ranked vignettes as $Vignette\,1 \leq Vignette\,\Gamma \leq Vignette\,4 \leq Vignette\,5$ with $\Gamma = \{2, 3\}$. 16.5% of the respondents have one violation of this order and 14.6% of respondents have more than one violation. It is worth noting that about 1.5% of the respondents violated all the possible rankings of the vignettes, probably because they misunderstood the VAS scale (reverse order).

Since these violations might just be measurement errors, it is informative to evaluate the size of these inconsistencies. We define the size of these violations as the average distance between vignettes that were incorrectly ranked and their corresponding reference point. For instance, if a respondent gives a VAS score of 60 to vignette 1, and a score of 30, 40, 50 and 80 for vignettes 2, 3, 4 and 5, respectively, then the average size of the violations would be 20. Indeed, the evaluation of vignette 1 by that respondent generates three violations (with vignettes 2, 3 and 4). The size of these violations are 30, 20 and 10 for vignettes 2, 3 and 4, respectively. Taking the sum of these distances (60) and dividing by the number of violations (3) gives the score of 20. The figure below displays the density of this measure for respondents who have a positive number of violations. The plot indicates that most violations are small in size (mean = 15.6) and therefore that violations are likely caused by measurement errors.

Finally, the table below provides estimates of our double-index model specification keeping in our sample only respondents with "consistent" ranking of the vignettes ($Vignette\,1 \leq Vignette\,\Gamma \leq Vignette\,4 \leq Vignette\,5$ with $\Gamma = \{2, 3\}$), that is dropping respondents who have at least one violation. As evidenced in the table below, the results are very similar to the ones we obtained in our benchmark results. That is, there exists some reporting heterogeneity in self-assessed sleep quality that is driven mainly by individuals who suffer from moderate depressive symptoms. Indeed, respondents with moderate depressive symptoms perceive the VAS scale differently from others, with their sleep difficulty score being 3.7-4 points higher than what it "really should be". Note however that this effect is small in magnitude, and that the associations between sleep difficulties and depressive symptoms remain very strong even after controlling for reporting heterogeneity, in line with our benchmark results.

[INSERT FIGURE S4 HERE] **Vignette equivalence**

Note: We define the size of ranking violations as the average distance between vignettes that were incorrectly ranked. See text for more details.

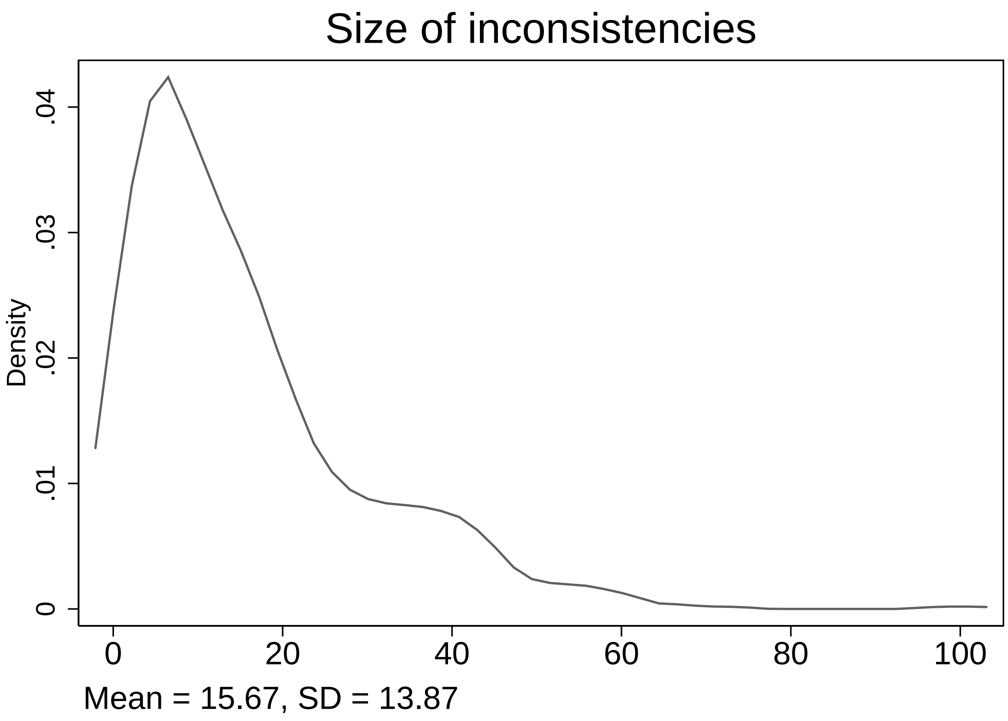

Ranking of vignettes with violations

Number of violations in vignette ranking	Cases	Proportion	Cumulative proportion
0	1249	68.89	68.89
1	299	16.49	85.38
2	118	6.51	91.89
3	40	2.21	94.10
4	21	1.16	95.26
5	22	1.21	96.47
6	17	0.94	97.41
7	13	0.72	98.12
8	7	0.39	98.51
9	27	1.49	100.00
Total	1813	100.00	

Note: The consistent vignette ranking is defined as: $Vignette\,1 \leq Vignette\,\Gamma \leq Vignette\,4 \leq Vignette\,5$ with $\Gamma = \{2, 3\}$. Number of violations of this order is recorded.

Linear regressions of sleep difficulties on our set of control variables, accounting for reporting heterogeneity.

	(1) "True" effect b_0	(2) Reporting Heterogeneity γ	(3) "True effect" b_0	(4) Reporting Heterogeneity γ
Female	2.60*	-0.33	1.17	-0.29
	(1.46)	(0.60)	(1.31)	(0.61)
Age	0.028	0.032	-0.078	0.042
	(0.47)	(0.20)	(0.44)	(0.20)
Mild depressive symptoms	21.3***	0.030	15.3***	0.22
	(2.12)	(0.82)	(1.96)	(0.83)
Moderate depressive symptoms	28.3***	3.71**	18.7***	4.05***
	(3.14)	(1.47)	(2.79)	(1.50)
Severe depressive symptoms	32.7***	3.49	26.3***	3.75
	(4.67)	(2.27)	(4.11)	(2.28)
Take more than 20 mins to fall asleep			18.7***	-0.43
			(1.44)	(0.63)
Sleep less than 7 hours			6.73***	-0.20
			(1.79)	(0.75)
Low sleep efficiency			9.50***	-0.64
			(1.89)	(0.82)
Constant	14.6		12.1	
	(11.3)		(10.7)	

Note: Cluster robust standard errors at the respondent level reported in parentheses (* $p < 0.1$, ** $p < 0.05$, *** $p < 0.01$). We also control for parent's income, education level, nationality, number of siblings, relationship status, relationship with parents, number of close friends, and school performance. These coefficients are not reported in the table but are available upon request. Columns 1 and 3 show the effects of the control variables on sleep difficulties, net of reporting heterogeneity, while columns 2 and 4 show the effects of the control variables on reporting heterogeneity.
