# Supplementary material for: The association between depressive symptoms and self-reported sleep difficulties among college students: Truth or reporting bias?
Source: PLoS One. 2021 Feb 19;16(2):e0246370. doi: 10.1371/journal.pone.0246370 (PMC7894923; doi:10.1371/journal.pone.0246370)
Supplement: S5 Appendix — (PDF) [file pone.0246370.s006.pdf]

S5 Appendix. Test of linear specification and fractional probit regression

While the linear model specification is simple and easy to implement/interpret, it may suffer from misspecification. We implement a number of tests and robust estimations to safeguard our assumption of linear regression models. First, we use individual-clustered variance estimation to account for heteroskedasticity. Second, we use regression specification-error test (RESET) to examine the linear assumption in the specification in which we regress sleep difficulty on our set of control variables (econometric model displayed in Table 2 column 1). The result of this test suggests no violation of the linear assumption ($F(3, 1783) = 1.24, p = 0.29$). Finally, given that our VAS measures are bounded between 0 and 100, we use fractional probit response estimator to estimate the regression of sleep difficulty on our control variables and compare the results with the corresponding linear specification estimates. As shown in the table below, the fractional probit model and linear model produce very similar estimates.

Linear model and fractional probit model

	Linear model	Fractional probit model
Female	1.24	1.25
	(1.20)	(1.19)
Age	0.20	0.18
	(0.39)	(0.39)
Mild depressive symptoms	19.6***	19.6***
	(1.69)	(1.68)
Moderate depressive symptoms	32.4***	32.4***
	(2.40)	(2.41)
Severe depressive symptoms	37.4***	37.6***
	(3.69)	(3.80)
Constant	17.5*	
	(9.93)	

Note: Robust standard errors are in parentheses (* $p < 0.1$, ** $p < 0.05$, *** $p < 0.01$). For fractional probit model, the marginal effects are reported. We also control for parent's income, education level, nationality, number of siblings, relationship status, relationship with parents, number of close friends and school performance. These coefficients are not reported in the table but are available upon request.
