# Supplementary material for: The association between depressive symptoms and self-reported sleep difficulties among college students: Truth or reporting bias?
Source: PLoS One. 2021 Feb 19;16(2):e0246370. doi: 10.1371/journal.pone.0246370 (PMC7894923; doi:10.1371/journal.pone.0246370)
Supplement: S1 Table — (PDF) [file pone.0246370.s007.pdf]

S1 Table. Descriptive statistics of sleep patterns

Descriptive statistics ($N = 1,813$)

Variable	Mean	SD	Min	Max
Hours of sleep (reported)	7.29	1.03	2	17
Hours of sleep (wake up time - bed time)	7.85	1.10	3.17	11
Hours of sleep, adjusted for time it takes to fall asleep	7.48	1.14	1.33	10.8
Time to fall asleep (in hours)	0.37	0.36	0	4.17
Take more than 20 mins to fall asleep	0.45	0.50	0	1
Sleep less than 7 hours	0.20	0.40	0	1
Low sleep efficiency	0.17	0.38	0	1

Note: Unweighted sample characteristics of the students who have completed our online survey in April 2017.
