# Supplementary material for: The association between depressive symptoms and self-reported sleep difficulties among college students: Truth or reporting bias?
Source: PLoS One. 2021 Feb 19;16(2):e0246370. doi: 10.1371/journal.pone.0246370 (PMC7894923; doi:10.1371/journal.pone.0246370)
Supplement: S2 Table — (PDF) [file pone.0246370.s008.pdf]

S2 Table. PHQ-9 as a continuous measure

Linear regressions of sleep difficulties on our set of control variables.

	(1) Sleep difficulty	(2) Sleep difficulty	(3) Sleep difficulty	(4) Sleep difficulty	(5) Sleep difficulty	(6) Sleep difficulty
Female	-0.41	-0.0033	-0.37	-0.025	-0.50	-1.01
	(1.15)	(1.14)	(1.16)	(1.15)	(1.10)	(1.06)
Age	0.30	0.19	0.26	0.29	0.20	0.22
	(0.38)	(0.37)	(0.38)	(0.38)	(0.37)	(0.35)
PHQ-9 score	2.95***	2.77***	2.94***	2.86***	2.32***	2.28***
	(0.14)	(0.13)	(0.14)	(0.14)	(0.15)	(0.13)
Hours of sleep (reported)		-4.56***				
		(0.59)				
Hours of sleep (wake up time - bed time)			-0.46			
			(0.54)			
Hours of sleep, adjusted for time it takes to fall asleep				-2.44***		
				(0.53)		
Time to fall asleep (in hrs)					23.1***	
					(2.61)	
Take more than 20 mins to fall asleep						16.4***
						(1.14)
Sleep less than 7 hours						5.41***
						(1.38)
Low sleep efficiency						8.56***
						(1.43)
Constant	1.87	39.6***	6.51	20.8**	-1.84	0.13
	(9.82)	(10.9)	(10.6)	(10.5)	(9.80)	(9.10)

Note: Robust standard errors in parentheses (* $p < 0.1$, ** $p < 0.05$, *** $p < 0.01$). We also control for parent's income, education level, nationality, number of siblings, relationship status, relationship with parents, number of close friends and school performance. These coefficients are not reported in the table but are available upon request.

Linear regressions of vignette evaluations on our set of control variables.

	(1) Vignette 1	(2) Vignette 2	(3) Vignette 3	(4) Vignette 4	(5) Vignette 5
Female	2.34**	0.15	-1.56*	-0.90	-1.64*
	(0.97)	(1.24)	(0.85)	(0.97)	(1.00)
Age	0.22	0.35	-0.25	-0.25	-0.27
	(0.31)	(0.39)	(0.28)	(0.31)	(0.31)
PHQ-9 score	0.23*	0.55***	0.070	-0.011	-0.15
	(0.12)	(0.15)	(0.10)	(0.11)	(0.12)
Constant	1.53	34.7***	66.4***	85.5***	96.1***
	(7.96)	(10.1)	(7.38)	(7.95)	(8.04)

Note: Robust standard errors in parentheses (* $p < 0.1$, ** $p < 0.05$, *** $p < 0.01$). We also control for parent's income, education level, nationality, number of siblings, relationship status, relationship with parents, number of close friends and school performance. These coefficients are not reported in the table but are available upon request.

Linear regressions of sleep difficulties on our set of control variables, including the vignette evaluations in columns 2 and 4.

	(1) Sleep difficulty	(2) Sleep difficulty	(3) Sleep difficulty	(4) Sleep difficulty
Female	-0.41	-0.53	-1.01	-1.12
	(1.15)	(1.15)	(1.06)	(1.05)
Age	0.30	0.28	0.22	0.21
	(0.38)	(0.38)	(0.35)	(0.35)
PHQ-9 score	2.95***	2.89***	2.28***	2.21***
	(0.14)	(0.14)	(0.13)	(0.13)
Vignette 1		0.093***		0.098***
		(0.034)		(0.031)
Vignette 2		0.073***		0.075***
		(0.025)		(0.023)
Vignette 3		0.028		0.044
		(0.039)		(0.034)
Vignette 4		0.083*		0.078**
		(0.043)		(0.038)
Vignette 5		-0.0062		-0.0018
		(0.043)		(0.040)
Take more than 20 mins to fall asleep			16.4***	16.5***
			(1.14)	(1.13)
Sleep less than 7 hours			5.41***	5.49***
			(1.38)	(1.37)
Low sleep efficiency			8.56***	8.64***
			(1.43)	(1.43)
Constant	1.87	-9.15	0.13	-12.0
	(9.82)	(10.3)	(9.10)	(9.63)

Note: Robust standard errors in parentheses (* $p < 0.1$, ** $p < 0.05$, *** $p < 0.01$). We also control for parent's income, education level, nationality, number of siblings, relationship status, relationship with parents, number of close friends and school performance. These coefficients are not reported in the table but are available upon request.

Linear regressions of sleep difficulties on our set of control variables, accounting for reporting heterogeneity.

	(1)<br>"True effect"<br>b_0	(2)<br>Reporting Heterogeneity<br>γ	(3)<br>"True effect"<br>b_0	(4)<br>Reporting Heterogeneity<br>γ
Female	-0.088	-0.32	-0.71	-0.30
	(1.21)	(0.56)	(1.12)	(0.56)
Age	0.34	-0.039	0.27	-0.045
	(0.41)	(0.19)	(0.38)	(0.19)
PHQ-9 score	2.81^{***}	0.14^{**}	2.13^{***}	0.15^{**}
	(0.15)	(0.069)	(0.14)	(0.073)
Take more than 20 mins to fall asleep			17.1^{***}	-0.67
			(1.19)	(0.57)
Sleep less than 7 hours			5.23^{***}	0.18
			(1.46)	(0.71)
Low sleep efficiency			8.61^{***}	-0.049
			(1.56)	(0.75)
Constant	1.87	1.87	0.13	0.13
	(9.78)	(9.78)	(9.06)	(9.06)

Note: Cluster robust standard errors at the respondent level reported in parentheses (* $p < 0.1$, ** $p < 0.05$, *** $p < 0.01$). We also control for parent's income, education level, nationality, number of siblings, relationship status, relationship with parents, number of close friends, and school performance. These coefficients are not reported in the table but are available upon request. Columns 1 and 3 show the effects of the control variables on sleep difficulties, net of reporting heterogeneity, while columns 2 and 4 show the effects of the control variables on reporting heterogeneity.
