# Supplementary material for: The association between depressive symptoms and self-reported sleep difficulties among college students: Truth or reporting bias?
Source: PLoS One. 2021 Feb 19;16(2):e0246370. doi: 10.1371/journal.pone.0246370 (PMC7894923; doi:10.1371/journal.pone.0246370)
Supplement: Attachment — Submitted filename: Response reviewers.docx [file pone.0246370.s009.docx]

# Response letter to the reviewers’ comments on manuscript submission PONE-D-20-28953 entitled “The Association Between Depressive Symptoms and Self-reported Sleep Difficulties Among College Students: Truth or Reporting Bias?”

Dear Editor,

dear Reviewers:

Thank you very much for your valuable feedback on our manuscript **PONE-D-20-28953**. We appreciate the opportunity to revise and resubmit our manuscript to *PLOS ONE*. The provided comments raised some good points and were helpful to improve the manuscript. We hope that the revised paper will be acceptable for publication in *PLOS ONE.*

This letter outlines our changes in the manuscript in response to the reviewers’ comments and suggestions and provides specific answers to all issues raised in their reviews. For convenience, we first reproduce the reviewers’ comments and then provide corresponding answers after each comment in italics.

**Reviewers' comments:**

*Reviewer #1:*

Overall assessment: Strengths include the large sample size and robust vignette analyses. While the manuscript has a generally logical flow, it carries an inefficient writing style. Prose is wandering throughout and flawed at times. Use of a non-clinical sample is not consistently conveyed when framing the study’s purpose. The finding of response heterogeneity in the depressed group is an interesting one, which supports the negative bias hypothesis. Yet, this was not fully explored in the discussion. I also wonder if mediator/moderator relationships are possible given the vignette findings--an area for potential consideration and exploration.

*Authors’ response:*

*Thank you very much for your very constructive comments. We revised our manuscript along the lines you suggest,*

*and we believe our paper is now much stronger than it was before. Thanks to your comments, the writing style is*

*more efficient. Importantly, we clarified that our study sample	does not only include college students with*

*depressive symptoms but that students with no or minimal symptoms are also included in the analysis. In fact,*

*they are the reference group in all	our analysis. We also expanded the discussion section, which now contains a*

*more in-depth discussion about the implications of the presence of reporting heterogeneity in sleep*

*difficulties among individuals with depressive symptom, albeit rather small.*

Abstract

1) “reliable” does not fit in the context of a significant association between vignette responses and depressive

symptomatology

*Authors’ response:*

*Thank you for this comment. We meant to say that “unadjusted” comparisons of self-reported sleep difficulties*

*among college students can be trusted because our analysis does not detect the presence of large reporting*

*heterogeneity between individuals with and without depressive symptoms. That’s the reason why we wrote*

*in the previous version of the abstract that comparisons are “reliable”. We do understand that it might not be*

*entirely appropriate, however. We have therefore changed the last sentence of the abstract, which now reads*

*as follows:*

*“This suggests that unadjusted comparisons of self-reported sleep difficulties between college students are*

*meaningful, even among individuals with depressive symptoms.”*

Data Availability

2) Where have data been made available, as indicated in your statement? The instructions request completed

sentences here.

*Authors’ response:*

*We have included the data and the codes as Supporting Information files in our resubmission.*

Introduction

3) Line 13: Omitting the phrase, “making the college student population vulnerable to mental health problems,”

would more succinctly flow with the supportive points that follow. As is, it feels like the cart is before the horse.

*Author’s response:*

*Thank you for your suggestion. We removed the phrase following your suggestion.*

4) Lines 19-21: Numbers for statistics and for citations are confusing due to dashes here, which seem misformatted.

This is also part of a lengthy sentence that could better connect the ideas in this paragraph and next with smaller

phrases.

*Author’s response:*

*You are absolutely right. We have in fact remove the sentence “75% of individuals who are diagnosed with a*

*mental health disorder throughout their life have had their first onset by the age of 25”, which didn’t add much*

*substance to the argument and made the sentence very long.*

5) Lines 39-43: To indicate discrepancy among these articles warrants, even if very brief, mention of possible or.

hypothesized explanation for these different findings.

*Author’s response:*

*We added a couple of sentences about possible or hypothesized explanations for these findings whenever it is*

*possible. Specifically, we state that:*

*“It has been shown that there exist significant differences between objective and subjective measures of sleep*

*difficulties [26, 36-40]. Buysse et al. (2008) have attributed the weak association between subjective and objective*

*measures of sleep to whether one assesses habitual patterns of sleep versus sleep on a discrete occasion. Baker et al.*

*(1999) have argued that the weak correlation between objective and subjective measures are due to between-*

*individual variation while Jean-Louis and Kripke (2000) have speculated that the subjective measures may be*

*biased by the depressive mood of respondents.”*

6) The introduction outlines the risk of mental health in adolescence but revision to the final paragraphs is first

needed to clearly link this idea to the present non-clinical sample. Importantly, lines 91-92 and 115 should be

corrected to indicate that participants consisted of both college students with and without elevated depressive

symptoms.

*Authors’ response:*

*You are right: lines 91-92 and 115 suggested that our study was exclusively based on a sample of*

*college students who suffer from depressive symptoms, whereas we do in fact compare the associations between*

*depression and sleep patterns for students with and without depressive symptoms (students without depressive*

*symptoms are the reference category in our analysis). This is a very important clarification to make and we thank*

*you for pointing that out.*

*We revised lines 91—92 as follows: “The objective of this study is to determine the existence of reporting*

*heterogeneity in subjective assessment of sleep difficulties among college students, and explore whether this*

*reporting heterogeneity is explained by differences in depressive symptoms.”*

*In the same vein, line 115 now reads as: “In addition, our study is the first to investigate the existence of*

*reporting heterogeneity in subjective assessment of sleep difficulties among college students, which, as*

*discussed above, is a strata of the population that is particularly prone to both sleep difficulties and mental*

*disorders.”*

*Hopefully these changes have made the link between the first and last part of the introduction clearer.*

7) Line 112: Both a dash and comma are not needed here, and in fact omitting “-a continuous measure-,” would

be more succinct.

*Authors’ response:*

*We made the change, thank you.*

Methods

8) Sparing use of footnotes is preferable, as I do not see a clear need for using footnotes in the sections before and

after but most notably in the methods (lines 121, 126, 133, 218). This information would be more efficient for

readers if integrated within the body of the text.

*Authors’ response:*

*Thank you for your comment. You are correct. In fact, PLOS ONE does not allow the use of footnotes. We have*

*therefore adapted our manuscript accordingly.*

9) Lines 131-132: The phrase “that generate major changes both in terms of mental health and sleep

patterns/circadian rhythm” warrants an addition citation. Consider rephrasing in a way that indicates a risk factor

rather than a definitive causal link if no firm citation is provided/available.

*Authors’ response:*

*We added a few citations that support our statement and replaced the word “generate” with “coincide with”*

*to avoid definitive causal link.*

10) Lines 137 & 139: should be “asked”

*Authors’ response:*

*We made the change, thank you.*

11) Line 206: “objective measures of sleep patterns” may lead readers to believe polysomnography or

actigraphy is in use rather than vignettes.

*Authors’ response:*

*We meant “objective measures of sleep patterns” as measures that are derived using objective scales (such as hours*

*of sleep) and hence not subject to reporting heterogeneity. But we understand that readers might interpret*

*“objective measures of sleep patterns” as measures of sleep pattern that are not self-reported. We therefore*

*changed our sentence to make our point clearer. The corresponding sentence now reads as follows:*

*“We further investigate the relationship between sleep difficulties and depressive symptoms using	multivariate*

*linear regressions, including various socio-demographic characteristics and self-reported measures of sleep*

*patterns that are not subject to reporting heterogeneity, e.g. hours of sleep as control variables.”*

Results

12) Given report here that 25% of the sample had scores on the depressive screener reaching clinically significant

levels, framing this sample as “depressed” here and elsewhere in the paper would seem confusing.

*Authors’ response:*

*We changed the introduction along the lines you suggested so that hopefully it is clear for the readers that our*

*sample include both students with and without depressive symptoms. The confusion was mainly coming from the*

*statement “among college students with depressive symptoms” which we now changed to “among college students”*

*in several instances in the paper.*

13) Were the assumptions of linear regression analyses met?

*Authors’ response:*

*We used linear models because it is simple and easy to implement/interpret. However, as you pointed out, it requires*

*a couple of assumptions. In the revision, to safeguard our assumption of linear regression models, we implemented*

*a number of tests and robust estimations, for which the corresponding results are presented in S5 Appendix.*

*Specifically, we first use individual-clustered variance estimation to account for heteroskedasticity to take into*

*account that we have several observations for each respondents (self-report and vignette evaluations). Second, we*

*use regression specification-error test (RESET) to examine the linear assumption in the specification in which we*

*regress sleep difficulty on our set of control variables (econometric model displayed in Table 2 column 1). The*

*result of this test suggests no violation of the linear assumption (F(3, 1783) =1.24 ,p=0.29)). Finally, given that our*

*VAS measures are bounded between 0 and 100, we use fractional probit response estimator to estimate the*

*regression of sleep difficulty on control variables and compare the results with the corresponding linear*

*specification estimates. As shown in the table of S5 Appendix, the fractional probit model and linear model produce*

*very similar estimates.*

*We added a sentence in the main text that refers to these results in S5 Appendix.*

14) Line 309: should be “the time it takes individuals”

*Authors’ response:*

*We made the change, thank you.*

15) Lines 323-236 and lines 400-102: Stating “do not appear to explain the relationship between depressive

symptoms and sleep difficulties” and “the associations between sleep difficulties and depressive symptoms

are unlikely to be severely biased” does not convey regression results truthfully. More accurate is the phrasing in

lines 232-335, highlighting that there remains to be a	significant association between sleep difficulty and depression

groups even after accounting for these other variables.

*Authors’ response:*

*What we meant by “do not appear to explain the relationship between depressive symptoms and sleep*

*difficulties” is that including the various measures of sleeping patterns and efficiency does not seem to*

*mediate/attenuate the associations between depressive symptom characteristics and self-assessed sleep*

*difficulties. We however understand the lack of clarity and have therefore modified the sentence accordingly,*

*which now reads as:*

*“Results show that including these three indicator variables in our statistical specification, although they are*

*individually all positive and highly significant (p<0.01), do not appear to attenuate the associations between*

*depressive symptoms and sleep difficulties.”*

*Regarding the second sentence you mention, we changed it to: “These results indicate that the associations*

*between sleep difficulties and depressive symptoms remain large and statistically significant even after controlling*

*for reporting heterogeneity.”*

*We hope these statements convey regression results more truthfully.*

16) Lines 327 and 426-427: Rather than “mental health” the emphasis should be placed on your analyzing the

PHQ-9 as a continuous measure vs. descriptive depression categories, and that findings were consistent.

*Authors’ response:*

*You are correct the sentences in lines 327 and 426-427 are confusing and we therefore changed them along the*

*lines you suggest. More specifically, the sentence that was in line 327 now reads as:*

*“Note that our results are robust to using PHQ-9 score as a continuous measure instead of descriptive*

*depression categories.”*

*The sentence that was in line 426-427 was replaced with:*

*“Again, these results are robust to using PHQ-9 score as a continuous measure instead of descriptive*

*depression categories (S2 Tables).”*

17) Line 407: “allows to” should be “allows us to”

*Authors’ response: We made the change, thank you.*

Discussion

18) Lacking is implications of results from the subset of the sample that carries severe depressive symptomatology,

which is the group most prone to negative bias in reporting.

*Authors’ response:*

*Thank you for this comment. Our results indicate that sleep difficulties of college students with moderate*

*depressive symptoms would be 2.6-2.7 points lower if they were to use the “same” reporting scale as college*

*students with no or minimal symptoms (reference group). As indicated	in our discussion section, given the*

*magnitude of the association between depressive symptoms and sleep difficulties, the magnitude of the difference*

*in reporting patterns between individuals with moderate symptoms and those with no or minimal symptoms*

*appear to be rather negligible. That being said, our analysis does not detect any differences in reporting*

*patterns between	individuals with no symptoms and those with severe depressive symptoms. So it does not seem*

*that individuals with severe depressive symptoms are the most prone to negative bias in reporting. We*

*modified the paragraph in the discussion section to make this point clearer.*

*A possible explanation for the absence of reporting heterogeneity among individuals with severe depressive*

*symptoms could stem from the fact that we have very few individuals in our sample that fall in that category*

*(1.8% of our sample). We mention that explanation in the discussion section as well.*

*Here is the paragraph that we updated in the discussion section:*

*“We show that college students who suffer from moderate depressive symptoms tend to be pessimistic about*

*their sleep difficulties, rating them 2.6-2.7 points higher than if they were to use the “same” reporting scale*

*as college students with no or minimal symptoms (reference group). Given the large magnitude of the associations*

*between sleep difficulties and depressive symptoms, the difference in reporting patterns between college*

*students with no or minimal symptoms and those with moderate depressive symptoms appear to 0	be minor (less*

*than 10% in magnitude). Interestingly, we do not find corresponding differences in reporting patterns*

*between individuals with no or minimal depressive symptoms and those with severe depressive symptoms.*

*Individuals with severe depressive symptoms appear to rate their sleep difficulties about 2 points higher than*

*if they were to use the same reporting scale as those with no or minimal symptoms, but this effect is not statistically*

*significant. A possible explanation	for this absence of reporting heterogeneity among individuals with severe*

*depressive symptoms could stem from the fact that we have very few individuals in our sample that fall in that*

*category (1.8% of our sample).”*

19) Line 433: Change to “college students asked to complete a survey...”

*Authors’ response:*

*We made the change, thank you.*

20) Lines 437-439: To be more succinct and avoid pitfalls described above, simplify this sentence	to reflect that

depression groups had greater sleep difficulty even after accounting for	differences in sleep patterns and efficiency.

*Authors’ response:*

*We change the text accordingly following your suggestion:*

*“Individuals with mild, moderate, and severe depressive symptoms rated their sleep difficulties to	be 20, 32, and 37*

*points higher, on a 0-100 Visual Analogue Scale, compared with those who have no or minimal depressive*

*symptoms. These associations hold even after accounting for differences in sleep patterns and efficiency.”*

21) Lines 441-444: omit “what it is when using the same reporting scale as” from this sentence, as	this is implied.

*Authors’ response:*

*We made the change, thank you.*

22) Lines 453-455 are the only reference to future research. What other areas for future study are implicated with

these findings?

*Authors’ response:*

*We expanded the discussion section by adding two sentences that discuss other research avenues that are*

*implicated with our findings. The sentences we added are the following:*

*“Moreover, our results indicate minor differences in reporting behaviors between individuals with and*

*without symptoms when it comes to evaluating their sleep difficulties. Future research could however explore the*

*presence of reporting heterogeneity across these groups in other domains such as self-reported quality of life and*

*wellbeing, for which corresponding reporting patterns are likely to be different.”*

23) Line 463: Again, “reliable” would be inaccurate given the significant response heterogeneity. Also change

“suffering from” to “with and without” or similar phrase.

*Authors’ response:*

*We changed the last paragraph of our manuscript following your suggestions. It now reads as follows:*

*Overall, the results of our study suggest that comparisons of self-assessed sleep difficulties between college students*

*are meaningful, even between those with and without depressive symptoms, and that reporting heterogeneity plays a*

*minor role in moderating the relationship between sleep difficulties and depressive symptoms.*

References:

24) Calling out citations mid-sentence with a bracketed number (e.g., lines 39, 40, 42, 108, 140, 151, footnote 10)

is less appealing than preceding this by an author name or a noun describing the	type of work. I would not be

surprised if this was inadvertently done by a citation manager.

*Authors’ response:*

*We agree that calling out citations mid-sentence with bracket numbers instead of the classic “author (year)”*

*format is suboptimal. We are using the PLOS ONE latex template which contains the “cite” package which*

*apparently does not allow us to straightforwardly call out both “[number]” and “author [number]” citations at the same time. To address your comment, we have manually added the name of the author(s) and year of the study we are citing when citations appear in the middle of the sentence.*

Tables/Figures:

25) Figures are missing y-axis labels.

*Authors’ response:*

*Sorry about this omission. The figures now contain the labels on the y-axis.*

26) The meaning of “vignette restricted” in the supplemental figures is not clearly described.

*Authors’ response:*

*Thank you for this comment. We added a short explanation in the text of the Appendix and in the notes of Figures*

*S3A and S3B. The notes of the figures now include the following sentence:*

*“The evaluations of the individuals who are included in that restricted sample are labeled “restricted'' in*

*the legend of the figure.” We hope that clarifies what we mean by “restricted”.*

27) Avoid using a form of the word “control” twice in titles (e.g., Table 5).

*Authors’ response:*

*We replaced “controlling” with “accounting” in Table 5 and in two tables in the Appendix.*

*Reviewer #2:*

1) The introduction is difficult to follow in that the author jumps around from mental health and sleep. While there are several variables (as examined in the methods/results) that may lead to depressive symptoms (such as family income and school performance), the PHQ9 does not assess any of those as they relate to depression. Furthermore, there is not sufficient discussion of the PHQ9 in the introduction to validate why it is the sole questionnaire being used to assess mental health in this study.

*Authors’ response:*

*We hope that the changes we made to the introduction thanks to reviewer 1 make it easier for readers to read the*

*introduction.*

*We opted for PHQ-9 to measure depressive symptoms of our respondents for several reasons. First, the PHQ-9 is a*

*well-validated instrument that is frequently used to screen for depression in primary care and non-clinical settings*

*(Arrieta et al., 2017; Kohler, Payne, Bandawe, & Kohler, 2017; Spitzer, Williams, & Kroenke, 2014). Second, PHQ-*

*9 has been shown to have good validity and credibility as an instrument for depression among college students*

*(Adewuya, Ola, & Afolabi, 2006; Huang, Kohler, & Kämpfen, 2020; Kim & Lee, 2019).*

*Finally, the use of the computerized version of the PHQ-9 has been validated, which is essential for our survey that*

*was conducted online (Erbe et al, 2016).*

*We added a sentence about PHQ-9 in the introduction, as well as more details in the “Measures on depressive*

*symptoms” subsection and acknowledged the limitations of the PHQ-9 instrument in the limitations.*

*Given the time and financial constraints we were facing in online survey, opting the PHQ-9 instrument was a very*

*natural choice to make. Nevertheless, we acknowledged the limitation of having used only one instrument to assess*

*the depressive symptoms of our respondents in the discussion. We added:*

*“Though widely used and validated, PHQ-9 is the sole instrument used in this study to determine the depressive*

*symptoms of our respondents and we therefore cannot rule out possible misclassification errors.”*

*References*

*Adewuya, A. O., Ola, B. A., & Afolabi, O. O. (2006). Validity of the patient health questionnaire (PHQ-9) as a screening tool for depression amongst Nigerian university students. Journal of Affective Disorders, 96(1–2), 89–93.*

*Arrieta, J., Aguerrebere, M., Raviola, G., Flores, H., Elliott, P., Espinosa, A., … others. (2017). Validity and utility of the Patient Health Questionnaire (PHQ)-2 and PHQ-9 for screening and diagnosis of depression in rural Chiapas, Mexico: A cross-sectional study. Journal of Clinical Psychology, 73(9), 1076–1090.*

*Erbe, D., Eichert, H. C., Rietz, C., & Ebert, D. (2016). Interformat reliability of the patient health questionnaire: validation of the computerized version of the PHQ-9. Internet Interventions, 5, 1-4.*

*Huang, Z., Kohler, I. V., & Kämpfen, F. (2020). A Single-Item Visual Analogue Scale (VAS) Measure for Assessing Depression Among College Students. Community Mental Health Journal, 56(2), 355–367. https://doi.org/10.1007/s10597-019-00469-7*

*Kim, Y. E., & Lee, B. (2019). The psychometric properties of the patient health questionnaire-9 in a sample of Korean university students. Psychiatry Investigation. https://doi.org/10.30773/pi.2019.0226*

*Kohler, I. V, Payne, C. F., Bandawe, C., & Kohler, H.-P. (2017). The demography of mental health among mature adults in a low-income, high-HIV-prevalence context. Demography, 54(4), 1529–1558.*

*Spitzer, R. L., Williams, J. B. W., & Kroenke, K. (2014). Test review: Patient Health Questionnaire--9 (PHQ-9). Rehabilitation Counseling Bulletin, 57(4), 246–248.*

2) Line 8: Give age range for “young adults”

*Authors’ response:*

*We added the age range for “young adults”.*

3) Line 11: Switching between “adolescence” and “young adults/college students” is confusing. I would decide on one term and be consistent with it. Also would advise defining what the age range is for which every population term you use

*Authors’ response:*

*Thank you for this comment. We used “young adults/college students” consistently in the revised manuscript. We*

*also added the age range when we referred to “young adults/college students” in the introduction, methods and*

*discussions.*

4) Lines 36-43: What are the objective measures you are referring to when comparing them with subjective measures?

*Authors’ response:*

*Polysomnographic recordings of sleep and actigraphy are the two usual techniques that are used to objectively*

*measure sleep quality. We now explicitly name these two techniques in the text, thank you.*

5) Line 117: There is no hypothesis/outcome measures stated for this study

*Authors’ response:*

*We added our hypothesis explicitly in the introduction section. We now state the following:*

*“We hypothesize that college students with and without depressive symptoms adopt different reporting scales when*

*assessing their sleep difficulties, to an extent that is hard to evaluate a priori.”*

Methods:

6) Of the 1813 respondents in your subject pool, did you only include those that recorded depressive symptoms on the PHQ9? Based on your description it is not clear. How was the Qualtrics survey worded when it was distributed? Without knowing this, it may lead a reader to wonder if there is non-respondent bias.

Authors’ response:

*We indeed included all students --with or without depressive symptoms-- in our study. As indicated in Table 1, 76%*

*of our 1813 respondents display no depressive symptoms. Students with no or minimal depressive symptoms are the*

*reference category in our analysis.*

*As pointed out by yourself and Reviewer1, the previous version of our manuscript did not make that clear enough*

*and we have revised our manuscript accordingly. Please see Reviewer 1 points 6 and 12 above.*

*Moreover, we added in the method section information about how students were recruited to participate in our*

*study. The sentence we added is the following:*

*“The invitation emails we sent to students and the introductory page of our online survey did not mention the*

*specific aim of the study but referred to it as a study on their quality of life in general.”*

*Finally, we can’t rule out the possibility that college students with depressive symptoms were less likely to*

*participate in our study and complete our survey. Selection bias can arise if, conditional on observables,*

*participants and non-participants are different in terms of sleep difficulties or reporting patterns. We acknowledged*

*this limitation in the discussion section.*

Overall comments:
I appreciate the idea behind this study but feel it is not framed in an appropriate way. It may be better written from a perspective of the self-reported sleep habit of individuals with and without depressive symptoms. As it reads now, it is unclear if the authors compared those with depressive symptoms to those without, which I feel is an important aspect to establish. Second, as mentioned in the Methods comments, there is no clear description of how the recruitment via Qualtrics was done. It should be made clear if this survey was sent out to students informing them it was for a study regarding depression and/or mental health as it relates to sleep. If this was in fact how it was stated, there is a risk of non-respondent bias in that students who experienced depression/mental health issue may have been more likely to respond to the survey than those that did not experience these symptoms. If the overall goal is to see if the reporting behaviors are different amongst individual with depressive symptoms then there should be 2 groups being compared (those with and those without).

*Authors’ response:*

*Thanks a lot for your positive assessment of our study and for your constructive comments. We hope our manuscript*

*is now clearer and the information provided regarding our study sample (which include both individuals with and*

*without depressive symptoms) and the recruitment procedure is more complete and better communicated.*

Journal’s comments:

1. Please ensure that your manuscript meets PLOS ONE's style requirements, including those for file naming. The PLOS ONE style templates can be found at

https://journals.plos.org/plosone/s/file?id=wjVg/PLOSOne_formatting_sample_main_body.pdf and

https://journals.plos.org/plosone/s/file?id=ba62/PLOSOne_formatting_sample_title_authors_affiliations.pdf

*Authors’ response:NA*

2. In your Data Availability statement, you have not specified where the minimal data set underlying the results described in your manuscript can be found. PLOS defines a study's minimal data set as the underlying data used to reach the conclusions drawn in the manuscript and any additional data required to replicate the reported study findings in their entirety. All PLOS journals require that the minimal data set be made fully available. For more information about our data policy, please see http://journals.plos.org/plosone/s/data-availability.

Upon re-submitting your revised manuscript, please upload your study’s minimal underlying data set as either Supporting Information files or to a stable, public repository and include the relevant URLs, DOIs, or accession numbers within your revised cover letter. For a list of acceptable repositories, please see http://journals.plos.org/plosone/s/data-availability#loc-recommended-repositories. Any potentially identifying patient information must be fully anonymized.

Important: If there are ethical or legal restrictions to sharing your data publicly, please explain these restrictions in detail. Please see our guidelines for more information on what we consider unacceptable restrictions to publicly sharing data: http://journals.plos.org/plosone/s/data-availability#loc-unacceptable-data-access-restrictions. Note that it is not acceptable for the authors to be the sole named individuals responsible for ensuring data access.

We will update your Data Availability statement to reflect the information you provide in your cover letter.

*Authors’ response:*

*We have uploaded the data we used in our study as well as the corresponding codes as Supporting information files.*

3. Please include a separate caption for each figure in your manuscript.

*Authors’ response:*

*We have included a caption for each figure These captions are in bold and are located right after the [INSERT*

*FIGURE XX HERE] in the tex file. Thank you.*
