# Supplementary material for: The association between depressive symptoms and self-reported sleep difficulties among college students: Truth or reporting bias?
Source: PLoS One. 2021 Feb 19;16(2):e0246370. doi: 10.1371/journal.pone.0246370 (PMC7894923; doi:10.1371/journal.pone.0246370)
Supplement: Attachment — Submitted filename: R2_Response reviewers.docx [file pone.0246370.s010.docx]

# Response letter to the reviewer’s comments on manuscript submission PONE-D-20-28953R1 entitled “The Association Between Depressive Symptoms and Self-reported Sleep Difficulties Among College Students: Truth or Reporting Bias?”

Dear Editor,

dear Reviewer:

Thank you again for your valuable feedback on our manuscript **PONE-D-20-28953R1**. We really appreciate the time Reviewer 1 has taken to provide us with her valuable feedback and comments that greatly improved the readability and flow of our manuscript. We hope that the revised paper will be acceptable for publication in *PLOS ONE.* This letter outlines our changes in the manuscript in response to Reviewer 1’s comments and suggestions and provides specific answers to all issues raised in her reviews. For convenience, we first reproduce Reviewer 1’s comments and then provide corresponding answers after each comment in italics.

**Reviewer's comments:**

*Reviewer #1:*

Methods

1)	-Line 134: Consider removing "against payment" or clarifying. If there is a lottery, I assume that not all students will be guaranteed renumeration.

*Authors’ answer: We removed “against payment”. You are correct: not all students were remunerated for their participation.*

2)	-Lines 159-162: This sentence could be shortened. For example, "This age restriction also corresponds to late adolescent/young adulthood [69], which represents..."

*Authors’ answer: Done, thank you!*

3)	-Lines 163-164: Removing the sentence "This adolescent/young adulthood age period is highly coincidental with the age distribution of our sample of college students," could make for a more succinct paragraph without loss of information.

*Authors’ answer: You are correct. That sentence was superfluous and didn’t provide additional information.*

4)	-Line 168: Change "sample consisted in" to "sample consisted of..."

*Authors’ answer: Done!*

5)	-Line 215: There is an extra space; please correct to "points."

*Authors’ answer: Done!*

6)	-Lines 230 & 231: Should be "As a robustness...as a measure..."

*Authors’ answer: Done!*

7)	-Line 232: Clarify what is meant by "alternative specification." The authors could rephrase to, "We show that our results when analyzing the presence of depressive symptoms are similar when analyzing depressive symptom severity," if this is accurate.

*Authors’ answer: Yes, this is accurate. We indeed meant that our results are robust to using either PhQ-9 as a continuous or as a categorical variable. We changed our sentence accordingly.*

8)	-Lines 253-257: Could shorten this sentence to: "This assumes that respondents use the same reporting scale when evaluating their own sleep difficulties as the scale they use when evaluating the sleep difficulties of the person described in the anchoring vignettes."

*Authors’ answer: The second part of the original sentence was indeed redundant, and we therefore modified the text along the lines you suggest.*

9)	-Line 258: Should be "does not allow us to..."

*Authors’ answer: Done!*

10)	-Lines 259-270: I see an opportunity here for the authors to be more concise. For example: "Another way to disentangle reporting heterogeneity and sleep difficulties is to perform a double-index model [60]. This model exploits respondents' evaluations of sleep difficulties for themselves and for vignettes, assuming that individuals use the same reporting scales. One can control for reporting heterogeneity as one controls for individual fixed effects in panel data models (where several observations are recorded for the same individual over time). The advantage is that the model allows us to not only control for reporting heterogeneity but also estimate the characteristics of the individuals that drive reporting heterogeneity."

*Authors’ answer: Thank you for this suggestion. We modified the paragraph following your suggestion.*

Results
11)	-Lines 349 & 356: I recommend avoiding the word "association," which is vague, and "decreasing" to describe the coefficients, as it may be confusing if these coefficients reflect an increased risk of sleep difficulties. Consider rephrasing such as, "For instance, the increase in sleep difficulties for those suffering from severe (moderate) depressive symptoms, compared to those with no depressive symptoms, only changed from ### points (p<0.01) to ### (p<0.01)," if this is accurate.

*Authors’ answer: You are right: we should be clearer about the fact that the coefficients reflect changes relative to the reference group, that is individuals with no or minimal symptoms. We modified the two sentences on lines 349 and 359 following your suggestions. Thanks again.*

12)	-Line 361: should be "the time it takes individuals"

*Authors’ answer: Done!*

13)	-Line 469: Again, further explanation of the coefficients would seem warranted. Perhaps change to: "(with increases of 20.1, 29.8, and 35.3 points, respectively, compared to those with no depressive symptoms, all with p<0.01)"

*Authors’ answer: You are right. We should have explicitly mentioned that the effects (coefficients) were relative to the reference group (individuals with no depressive symptoms). We followed your suggestions and change the corresponding sentence accordingly.*
